# Appetite for a Foodborne Infection

**DOI:** 10.1371/journal.ppat.1005124

**Published:** 2015-09-24

**Authors:** Vern B. Carruthers

**Affiliations:** Department of Microbiology and Immunology, University of Michigan Medical School, Ann Arbor, Michigan, United States of America

Most people who ask about our research are somewhat aghast to hear that their neurons might be dens for a brain parasite obtained from cats or from eating contaminated meat. Although *Toxoplasma gondii*, or “Toxo” for short, can be an aggressive cause of birth defects, eye disease, or fatal neural syndrome in people with weak immunity, many of the 2 billion people infected, including 60 million Americans, don’t know they have the parasite. Nonetheless, the cumulative effects of this infection are coming to light, exposing a plethora of subtle yet startling potential consequences, ranging from altered personality and behavior to worsening of mental illnesses and memory loss. While such effects were unknown when I first started studying Toxo, it was clear to me then that a parasite capable of infecting so many people must have some clever tricks up its sleeves. I was bent on exposing its tactics, beginning with understanding how it invades our cells.

Watching Toxo plunge into a cell—an experience you, too, can enjoy via the internet (https://www.youtube.com/watch?v=WFy2OkYC-gs)—is both fascinating and a bit disturbing. The efficiency with which it invades a cell evokes thoughts of an agent that is highly evolved, well equipped, and a formidable adversary. Indeed, elegant studies by many dedicated researchers are revealing the complex machinery the parasite uses to enter cells. Within this arena, my lab identified a parasite cathepsin protease that contributes to invasion by activating proteins Toxo uses to recognize a target cell. Although this protease has a relatively small role in invasion, subsequent basic research on its functions is opening up entirely unforeseen avenues of discovery, along with new opportunities for treatment.

For starters, studying the protease helped reveal for the first time that Toxo has a complete digestive system, including an organelle that resembles a lysosome, the “stomach” of a cell. Although most other eukaryotic cells readily ingest and digest material from their environment, Toxo lives within what was thought to be an exiled compartment in infected cells. So, it wasn’t obvious why Toxo had a lysosome. Nonetheless, we reasoned that the parasite might need to obtain and degrade material from its host. Ensuing work confirmed that it has a pathway to “eat” proteins derived from infected cells. Additional studies also showed that giving the parasite a case of “indigestion” slows its replication and renders it more prone to immune clearance during the phases of infection when symptoms are seen. Although there is a need to improve therapies for the symptomatic phases, developing a first-of-its-kind treatment for the chronic brain stage is one of the greatest challenges in the field. In a step toward this goal, our latest work is revealing that the Toxo cathepsin protease is crucial to the parasite when it is hibernating in neurons and that inhibiting the enzyme effectively kills this stage.

Looking back, it was impossible to predict that our basic studies on Toxo cell invasion could lead to a drug target for the pervasive chronic infection. The chronic stage is notoriously difficult to study because of its slow growth, making it challenging to identify potential treatments in the absence of basic knowledge. Like sweet treats at the top of the food pyramid, virtually all new medicines rest upon foundational bricks of insight from basic research. Lacking a balanced diet replete with fundamental knowledge, the research pyramid loses structural integrity and gains unrealized possibilities. Like most basic researchers, I’m pushed by an appetite for discovery and pulled by a persistent desire to translate breakthroughs for improving human health. However, realizing this potential depends on securing a grant with an approximately 1 in 10 chance of approval with each submission. The parasite might be safe it its den (for now).

**Image 1:**
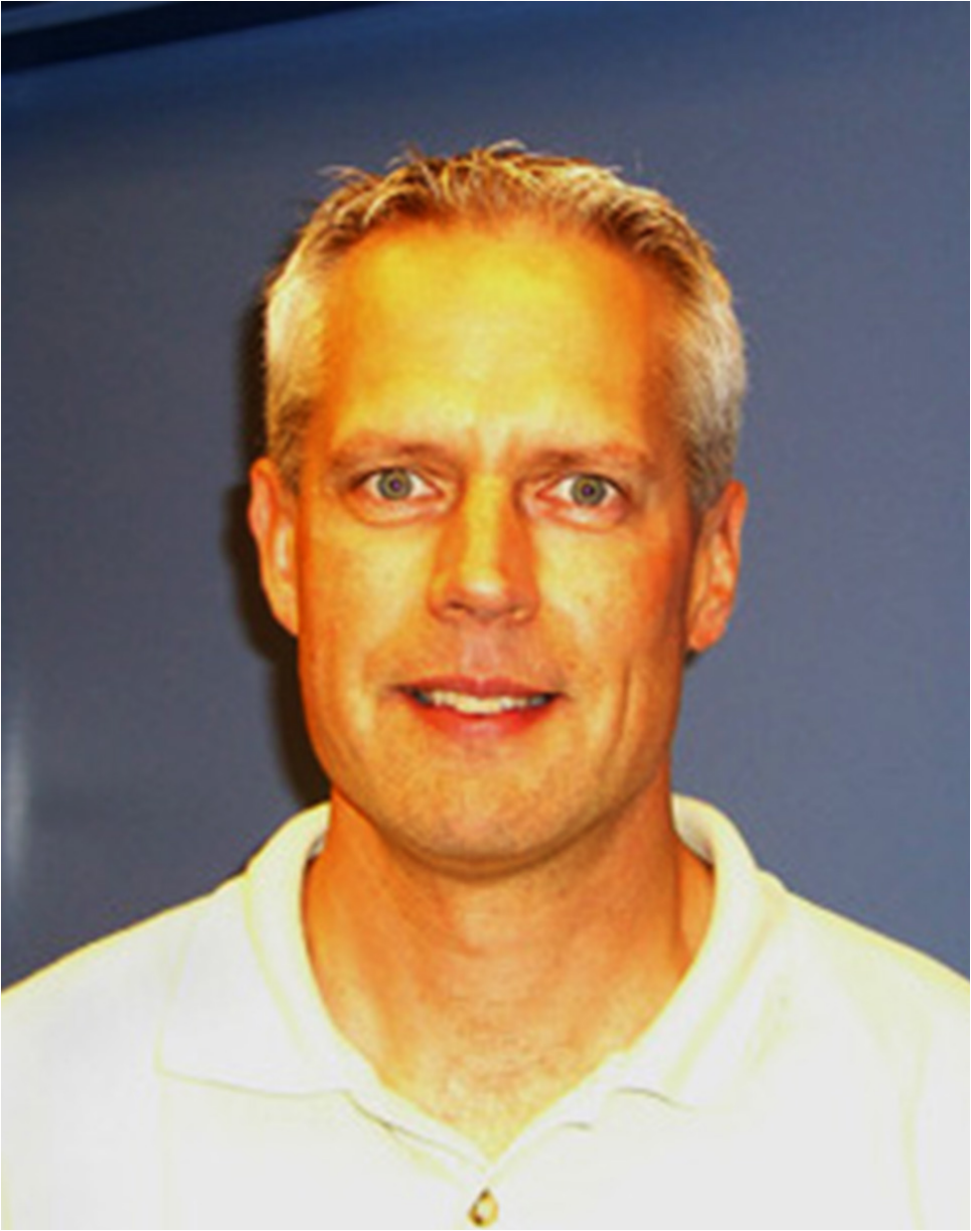
Vern B. Carruthers.

